# Developmental Link between Sex and Nutrition; *doublesex* Regulates Sex-Specific Mandible Growth via Juvenile Hormone Signaling in Stag Beetles

**DOI:** 10.1371/journal.pgen.1004098

**Published:** 2014-01-16

**Authors:** Hiroki Gotoh, Hitoshi Miyakawa, Asano Ishikawa, Yuki Ishikawa, Yasuhiro Sugime, Douglas J. Emlen, Laura C. Lavine, Toru Miura

**Affiliations:** 1 Graduate School of Environmental Science, Hokkaido University, Sapporo, Hokkaido, Japan; 2 Department of Entomology, Washington State University, Pullman, Washington, United States of America; 3 Okazaki Institute for Integrative Bioscience, National Institute for Basic Biology, National Institutes of Natural Sciences, Okazaki, Aichi, Japan; 4 Ecological Genetics Laboratory, Center for Frontier Research, National Institute of Genetics, Mishima, Shizuoka, Japan; 5 Graduate School of Life Sciences, Tohoku University, Sendai, Miyagi, Japan; 6 Division of Biological Sciences, The University of Montana-Missoula, Missoula, Montana, United States of America; University of California Davis, United States of America

## Abstract

Sexual dimorphisms in trait expression are widespread among animals and are especially pronounced in ornaments and weapons of sexual selection, which can attain exaggerated sizes. Expression of exaggerated traits is usually male-specific and nutrition sensitive. Consequently, the developmental mechanisms generating sexually dimorphic growth and nutrition-dependent phenotypic plasticity are each likely to regulate the expression of extreme structures. Yet we know little about how either of these mechanisms work, much less how they might interact with each other. We investigated the developmental mechanisms of sex-specific mandible growth in the stag beetle *Cyclommatus metallifer*, focusing on *doublesex* gene function and its interaction with juvenile hormone (JH) signaling. *doublesex* genes encode transcription factors that orchestrate male and female specific trait development, and JH acts as a mediator between nutrition and mandible growth. We found that the *Cmdsx* gene regulates sex differentiation in the stag beetle. Knockdown of *Cmdsx* by RNA-interference in both males and females produced intersex phenotypes, indicating a role for *Cmdsx* in sex-specific trait growth. By combining knockdown of *Cmdsx* with JH treatment, we showed that female-specific splice variants of *Cmdsx* contribute to the insensitivity of female mandibles to JH: knockdown of *Cmdsx* reversed this pattern, so that mandibles in knockdown females were stimulated to grow by JH treatment. In contrast, mandibles in knockdown males retained some sensitivity to JH, though mandibles in these individuals did not attain the full sizes of wild type males. We suggest that moderate JH sensitivity of mandibular cells may be the default developmental state for both sexes, with sex-specific Dsx protein decreasing sensitivity in females, and increasing it in males. This study is the first to demonstrate a causal link between the sex determination and JH signaling pathways, which clearly interact to determine the developmental fates and final sizes of nutrition-dependent secondary-sexual characters.

## Author Summary

Sexual dimorphisms such as the exaggerated antlers of deer, the enormous clawed chelae of crabs, and the horns and mandibles of beetles, are widespread across animal taxa and have fascinated biologists for centuries. Much recent work has uncovered the importance of the role of the sex-determination pathway in the expression of sexually dimorphic traits. However, critical interactions between this pathway and other growth regulatory mechanisms – for example, the physiological mechanisms involved in nutrition-dependent expression of these traits – are less well understood. In this study, we provide evidence of a developmental link between nutrition-sensitivity and sexual differentiation in the giant mandibles of the sexually dimorphic stag beetle, *Cyclommatus metallifer*. We examined the regulation and function of a key sex determination gene in animals, *doublesex* (*dsx*), and its interaction with juvenile hormone (JH), an important insect hormone known to regulate insect polyphenisms including the regulation of the disproportionate growth of male stag beetle mandibles. We found that *Cmdsx* changes mandibular responsiveness to JH in a sex-specific pattern. Based on these results, we hypothesize that sex-specific regulation of JH responsiveness is a developmental link between nutrition and sexual differentiation in stag beetles.

## Introduction

The evolution of sex-specific traits in animals has long fascinated biologists. How is growth regulated so that it differs dramatically between males and females? Sexual dimorphisms are widespread across diverse animal taxa and include exaggerated sexually selected traits like the antlers of deer, the enormous clawed chelae of crabs, and the elaborate trains of peacocks [1], [2], [3]. Some of the most striking sexually dimorphic traits are found within insects, such as the horns of rhinoceros beetles and the large mandibles of male stag beetles [3], [4], [5].

Sex-specific exaggerated traits often develop in a condition-dependent manner, so that not all individuals produce the trait even in the same sex [6], [7], [8], [9], [10]. Virtually all of the most extreme ornaments and weapons are also conditionally-expressed; they are exquisitely phenotypically plastic structures, whose growth depends on larval/juvenile access to nutrition [3], [5], [9], [11], [12]. Consequently, developmental mechanisms generating sex-specific trait growth and nutrition-dependent phenotypic plasticity are each likely to regulate the expression of extreme structures of sexual selection. The near universality of sex differences in the nutrition sensitivity of these traits suggests that common developmental mechanisms may be involved. Yet we still know almost nothing about how the processes of sex-specific growth and nutrition-sensitivity interact with each other to generate sexual dimorphism.

Recent studies in model organisms such as the fruit fly, nematode, medaka fish, and mouse, implicate a group of highly conserved proteins known as DM, or DNA binding motif proteins, as major effectors of sexual differentiation (recently reviewed in [13] and [14]. The fruit fly DM domain gene *Doublesex (dsx)* is conserved in structure and function in all insect species where it has been examined [2], [15], [16], [17], [18], [19], [20], [21], [22]. The *dsx* gene is transcribed in both sexes, but then differentially spliced to produce a male-specific or a female-specific mRNA (for review see [13]). These alternatively-spliced sex-specific transcripts code for a male (DsxM) or a female-specific (DsxF) protein [13]. Both types of Dsx proteins contain a zinc finger-like DNA binding domain called the DM domain [23], and act as transcriptional regulators responsible for sexual differentiation of tissues during development [1], [2], [24], [25]. For these reasons, *dsx* is a promising candidate for the regulation of sexual dimorphisms in the weapons of beetles. Indeed, recent published papers on *dsx* function in dung beetles (*Onthophagus taurus* and *O. sagittarius*) and rhinoceros beetles demonstrate a functional role for *dsx* in sex-specific growth of horns [20], [22], [26].

**Figure 1:**
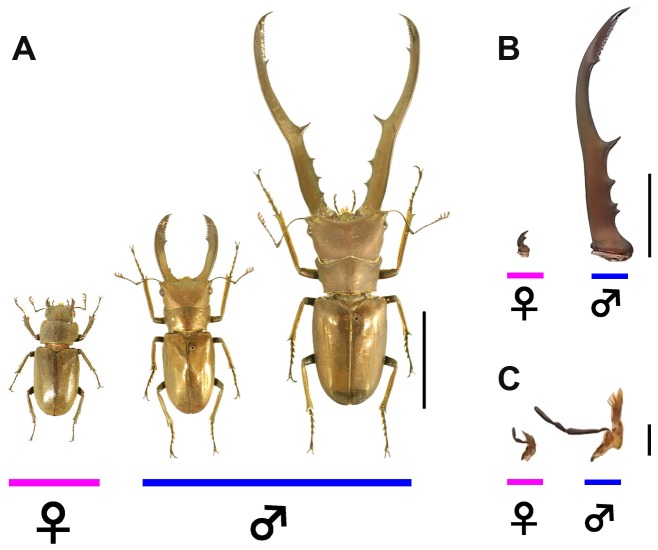
Focal stag beetle *Cyclommatus metallifer*. (**A**) Intraspecific sexual dimorphism and male variation in *Cyclommatus metallifer*. Female (left), small male (center), and large male (right) are shown. Scale bar indicates 20 mm. (**B**) This species exhibits strong sexual dimorphism of mandibles between the sexes. Mandibles of female (left) and large male (right) are shown. This difference in size is a result of male-specific disproportionate mandibular growth. Scale bar indicates 10 mm. (**C**) Maxilla are not sexually dimorphic. Maxilla of female (left) and large male (right) are shown. Scale bar indicates 2 mm.

In stag beetles, many species show strong sexual dimorphism in the size of their mandibles [5], [27], [28], [29], [30], [31], [32]. Males that have access to unlimited amounts of food as larvae develop disproportionately larger mandibles than males with restricted access to food, but more importantly, female mandibles never proliferate to the extent of even poorly-fed, small males (Fig. 1) [5]. Gotoh et al. recently found that nutrition-dependent mandibular growth in stag beetles is mediated by juvenile hormone (JH) in a sex-specific fashion [5]. JH titers were positively correlated with individual nutritional condition, and, in males, high JH titers promoted the growth of mandibles. In contrast, although females had similar levels of JH to males, female mandibles did not respond to high JH. Also, JH treatment did not affect to the growth of mandibles in females. These results indicate a sex-specific response of these traits to nutritional condition via JH [5]. However, other than this intriguing result, the mechanisms underlying developmental links between sex-determination, endocrine signaling, and sex-specific trait growth have yet to be characterized for any insects with exaggerated sexual dimorphism in insects.

**Figure 2:**
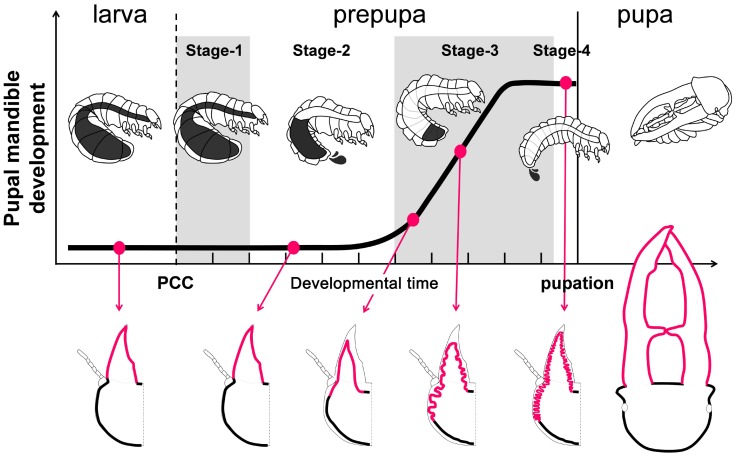
Developmental staging chart of prepupal development from the larval-prepupal transition to pupation. Mandibular growth (cell proliferation) is indicated on the y-axis over developmental time in days on the x-axis (hashmark = 1 day). Three distinct forms are recognized - the last instar larva, the prepupa, and the pupa. Known landmarks of mandibular proliferation in large males are indicated by the red circles and shown in diagram form below the graph [5]. The end of the larval period and the initiation of the prepupal period is indicated by Pupal Cell Construction (PCC) and is defined as the starting point of prepupal Stage 1. Outwardly the larva does not change its morphology. Stage 1 lasts approximately 2 days until the initiation of the first Gut Purge (GP) in which the prepupa begins to transform and empties out half of its gut contents. The time the prepupa spends in the first GP is known as Stage 2. Stage 3 is a quiescent phase where the prepupa undergoes massive adult imaginal tissue proliferation but outwardly appears suspended in the first GP. Stage 4 occurs over only a few hours and begins with the second GP in which the last remnants of the gut contents are egested and the prepupa completely metamorphoses into the pupa.

Here, we investigated the developmental mechanisms of sex-specific mandible growth in the stag beetle *Cyclommatus metallifer*, focusing on *dsx* gene function and its interaction with JH signaling. This species was used in previous studies on mandible development [5], [33] and we have recently constructed a transcriptome database for this species (Gotoh et al. in prep). To characterize *dsx* in *Cyclommatus metallifer*, the full length *C. metallifer dsx* (hereafter *Cmdsx*) transcript was obtained by degenerate PCR and subsequent Rapid Amplification of cDNA Ends PCR (RACE-PCR). Expression analyses of *Cmdsx* were carried out by Reverse Transcription PCR (RT-PCR) and real-time quantitative PCR (qPCR) to reveal the spatio-temporal expression pattern and sex-specificity of the *Cmdsx* transcripts during the prepupal period, which is known to be the critical period when mandibular tissues proliferate to their final adult size (Fig. 2) [5]. The function of *dsx* during sex-specific morphogenesis was investigated by gene knockdown using RNA interference (RNAi) against the *Cmdsx* transcripts. In addition to this, to investigate the putative interaction between *dsx* and endocrine (JH) signaling during mandibular growth, we ectopically applied JH analog to *dsx* ^RNAi^ individuals.

## Results

### Identification of sex-specific alternative splice variants of *Cyclommatus metallifer dsx*

**Figure 3:**
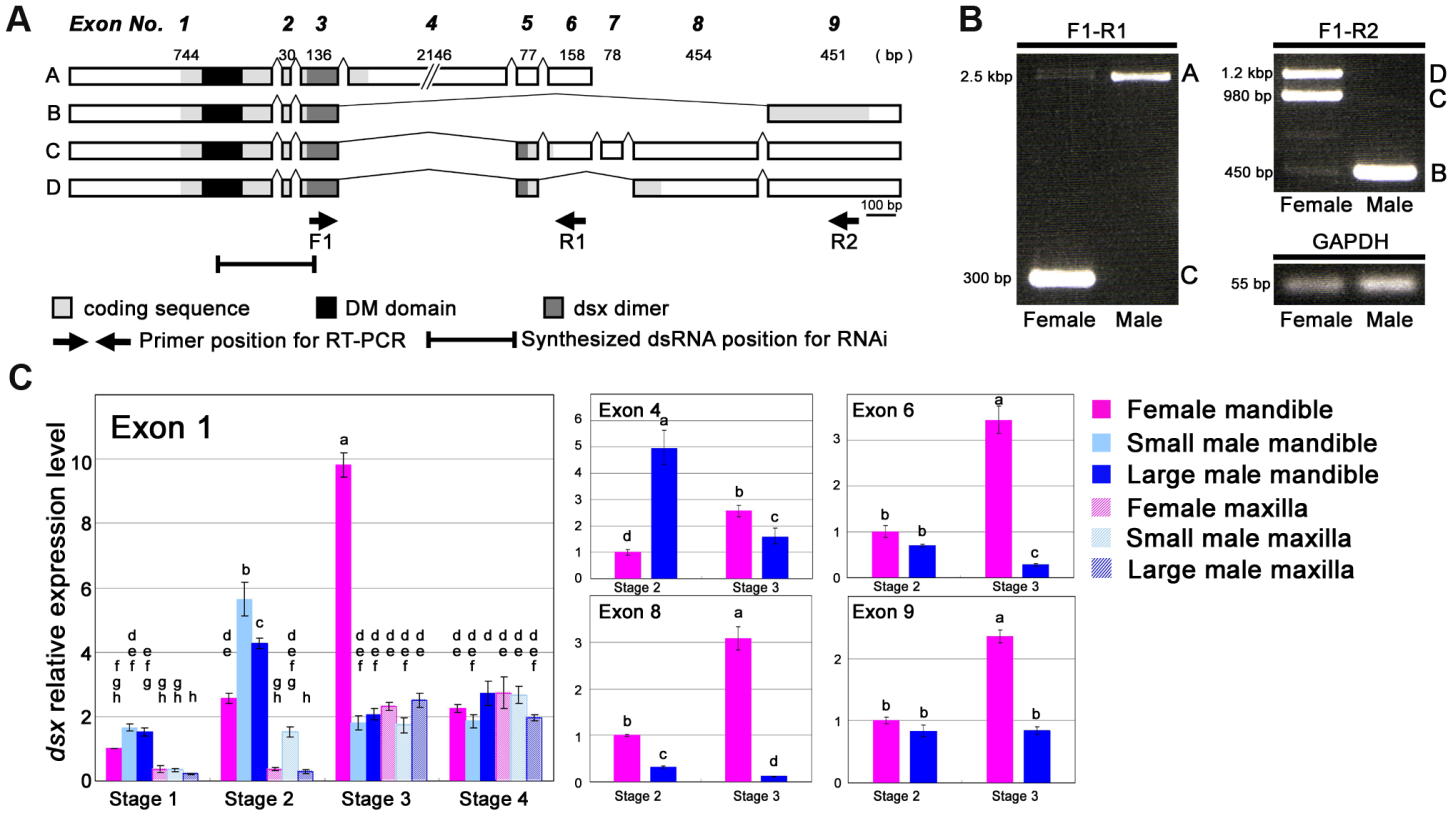
Characterization of the *Cyclommatus metallifer dsx* transcript. (**A**) Predicted gene models *C. metallifer doublesex* (*Cmdsx*) from transcripts. Four alternative splice variants were found and designated *Cmdsx* splice variants A, B, C and D. The coding sequence is in light gray, the conserved DM domain is in black, and the predicted dsx dimer formation site is in dark gray. Arrows indicate forward and reverse primer locations on the sequences and the region that was synthesized for dsRNA is indicated. (**B**) RT-PCR using exon-specific primers. Template cDNAs were derived from Stage 2 mandibles of both sexes. Transcripts A and B are male specific in expression and C and D are female specific in expression. (**C**) Temporal and spatial expression patterns of *Cmdsx* exon 1 in developing mandibular and maxillar tissues from small males, large males and females during the prepupal period. Total *Cmdsx* expression is shown by exon 1 as it is common to all four *Cmdsx* splice forms. Maxillae were used as a control trait as they show isometric growth both in males and females and do not show strong sexual dimorphism in *C. metallifer*. Relative expression of exons 4, 6, 8, & 9 are shown from Stage 2 male and female mandibles. The averages and 95% confidence intervals of three technical replicates are shown. For each exon, different small-case letters indicate significant differences (Tukey-Kramer test, P<0.05).

The full-length *Cmdsx* transcript was obtained by degenerate PCR and subsequent RACE-PCR (Fig. 3A). Four distinct splice variants (A, B, C and D isoforms) were identified, which contain the highly conserved DM domain and encode protein sequences with high sequence similarity to known insect Dsx proteins (Fig. S1, [34]). Protein sequence similarity of the stag beetle isoforms with that of the recently reported *doublesex* gene of *Onthophagus taurus* (Scarabaeidae, Coleoptera; [20]), and RT-PCR expression analyses show that *Cmdsx*A and *Cmdsx*B are male-specific, while isoforms *Cmdsx*C and *CmdsxD* are female-specific (Fig. 3B).

There are several differences in structure among the four splice variants of the *Cmdsx* transcript. First, a large exon (exon 4) containing primarily noncoding sequence and showing no similarity to *dsx* exons in any other insects, only occurs in splice variant A (Fig. 3A). In addition, *CmdsxA* contains neither exon 8 nor exon 9 (found in variants B, C, and D). *Cmdsx* variants C and D are similar to each other overall, with the exception of exon 6 which is only found in *CmdsxC* (Fig. 3A). An important difference between the predicted isoforms is the absence of 14 amino acid residues at the 3′-end of the conserved dsx dimer domain in the A and B isoforms (Fig. S1); this difference in the dsx dimer domain was also reported in the *doublesex* gene of *Onthophagus taurus* [20]. CmDsxB and the male-specific Dsx isoform of *O. taurus* (OtDsxM) also share a 25 amino acid sequence at their 3′ end (Fig. S1). Also CmDsxC and CmDsxD had similar 3′ end sequences to the female-specific Dsx isoforms of *O. taurus* (OtDsxF1 and OtDsxF2), respectively (Fig. S1).

### Sex- and tissue-specific expression of *dsx* during mandible growth

Expression patterns of exons of the *Cmdsx* transcript were examined in developing mandibles of both sexes in detail by real-time qRT-PCR during different stages of prepupal development (Fig. 3C) Tissue-specific expression and nutrition-dependent expression were examined by measuring the expression level of exon 1, which represents the total *Cmdsx* isoform expression since it is shared by all splice variants. Also, expression patterns of exons 4, 6, 8 and 9 were examined in order to characterize the sex-specific usage of these exons.

Mandibles are sexually dimorphic (e.g. males have disproportionately large mandibles, Fig. 1B) and were expected to show high *Cmdsx* expression. Maxillae, on the other hand, are not dramatically different in the two sexes (e.g. maxillae show isometric allometry in both sexes, Fig. 1C) and we expected lower levels of expression of *Cmdsx*. As predicted, expression of exon 1 was higher in mandibles than in maxillae in both males and females, especially during prepupal Stages 1 and 2 (Fig. 3C). Exon 1 was expressed at its highest level in male mandibles during prepupal Stage 2, but peaked later during (Stage 3) in female mandibles (Fig. 3C). No differences in expression of exon 1 were detected in Stage 4 of prepupal development in either trait (Fig. 3C).

Large and small males have different nutritional histories and undergo different amounts of mandible growth. However, expression levels of Exon 1 were similar for large and small males, during Stages 1, 3 and 4 in both mandibles and maxillae (Fig. 3C). Only during Stage 2 were there significant differences in expression of Exon 1 (Fig. 3C).

Sex-specificity of each exon was examined during Stages 2 and 3, when total *Cmdsx* expression reached its peak in males and females (Fig. 3C). Expression levels of Exon 4 were five times higher in males than females during Stage 2 (Fig. 3C) and only low levels of expression of this exon were found in both males and females during Stage 3 (Fig. 3C). The female-specific exon 8 was more highly expressed in females during both stages 2 and 3 but especially during Stage 3 (Fig. 3C). Exon 6 and exon 9 were expressed similarly in both males and females during Stage 2, but showed increased expression in females during Stage 3 (Fig. 3C). The increase of expression of exons 6 and 9 in Stage 3 females is expected to correspond to an overall expression peak of all *dsx* isoforms in females during Stage 3 (Fig. 3A)

### Functional analysis of *dsx* reveals a role in sex-specific mandible growth

**Figure 4:**
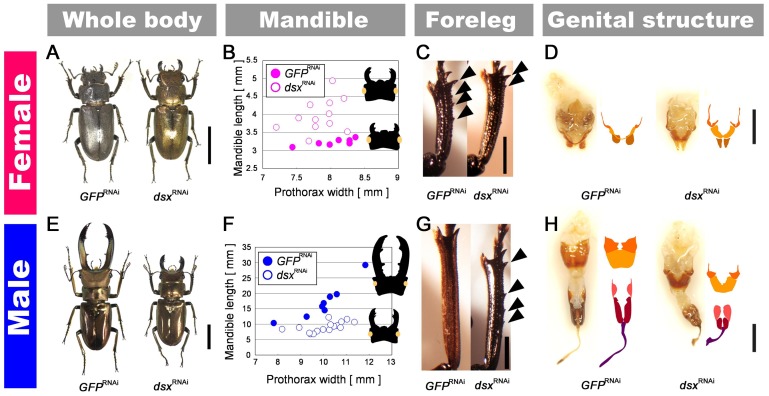
Intersex phenotypes of *dsx* ^RNAi^ in females and males. (**A, E**) Adult phenotypes of a *dsx* ^RNAi^ and control *GFP* ^RNAi^ individuals of both sexes. Scale bars indicate 10 mm. (**B, F**) The relationship between prothorax width (X-axis) and mandible length (Y-axis) for *GFP* ^RNAi^ individuals (closed circles) and *dsx* ^RNAi^ individuals (open circles) in females (pink) and males (blue). (**C, G**) Adult tibial phenotypes of *dsx* ^RNAi^ individuals. Arrowheads indicate female-specific tibial spines. Foreleg tibia of *GFP* ^RNAi^ female which has several female-specific spines. The foreleg tibia of a *dsx* ^RNAi^ female; note that the number of female-specific spines decreased in comparison to the *GFP* ^RNAi^ female. The foreleg tibia of a *dsx* ^RNAi^ male in which female-specific spines are seen. The foreleg tibia of a *GFP* ^RNAi^ male that does not show female-specific spines. Scale bars indicate 2 mm. (**D, H**) Adult genital phenotypes of *dsx* ^RNAi^ individuals. Dorsal view of genitalia of *GFP* ^RNAi^ female, *dsx* ^RNAi^ female, *dsx* ^RNAi^ male and *GFP* ^RNAi^ male. Schematic views of the genital plates are indicated next to the photographs. Genital plates that are homologous in males and females are indicated in the same color. Scale bars indicate 2 mm.

Injections of *Cmdsx* dsRNA reduced *Cmdsx* transcript abundances by 13–84% in prepupal mandibles, compared with control injections of *GFP* dsRNA (Fig. S2). The region of the *Cmdsx* transcript that was targeted extended from exon 1 to exon 3, and was therefore predicted to knockdown all four of the expressed transcripts of *Cmdsx* in both sexes (Fig. 3A). Knockdown of the *Cmdsx* gene by RNAi during prepupal development confirmed a significant functional role in the regulation of sex-specific mandible growth in stag beetles. The phenotype of *dsx* ^RNAi^ females was changed to be more male-like in body color, mandible size, foreleg tibial spine number, and genital shape and genital size (Fig. 4A, B, C, D). Significant mandible growth was induced in *dsx* ^RNAi^ females compared with *GFP* ^RNAi^ females (t = 4.509, *P* = 0.000357, Fig. 4B). In contrast, in *dsx* ^RNAi^ males, mandible growth was dramatically and significantly suppressed (Fig. 4E, F), resulting in more female-like forms. The relationships of body size and mandible size are significantly different between *GFP* ^RNAi^ and *dsx* ^RNAi^ males (F = 19.072, P = 0.0002982). In the range of the observed body size, *dsx* ^RNAi^ males possessed smaller mandibles, and the mandible-size difference became larger as body size increases (Fig. 4F). The intersex phenotypes resulting from the *dsx* ^RNAi^ knockdown extended to the body color of females, transforming them from the black color typical of females to a metallic copper typical of males (Fig. 4A). The number of spines on the tibia also changed. Females typically develop with four or five tibial spines (and males with zero). However, in *dsx* ^RNAi^ females this number decreased from four to fewer (becoming more male-like), and in *dsx* ^RNAi^ males the number of spines increased from zero to four (more female-like) (Fig. 4C, G). Finally, the size and length of the genitalia changed in sex-inappropriate directions in both *dsx* ^RNAi^ females and *dsx* ^RNAi^ males (Fig. 4D, H).

### Dsx modulates the response to JH in a sex-specific manner

We have previously shown that JH titer during the prepupal period is correlated with adult male body size and mandible size in the stag beetle, and that ectopic application of JH to the prepupal male induces male mandible proliferation [5]. At least part of the exquisite condition-sensitivity of extreme mandible growth appears to involve sensitivity of mandibular tissues to circulating JH. However, we have also shown that mandibular tissues of females do not respond to JH in the same way as males. Mandibles in females did not respond to ectopic JH, despite the fact that females had similar levels of circulating JH to males during this developmental period [5]. This suggests that female mandibular tissues may be insensitive to JH signaling.

**Figure 5:**
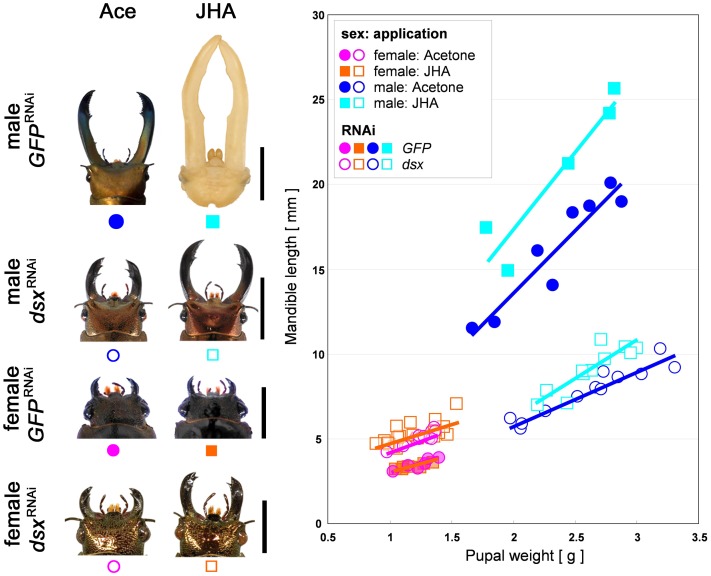
Effects of JHA application on *GFP* ^RNAi^ and *dsx* ^RNAi^ individuals. The relationships between pupal weight and mandible length were described. Sex is indicated by color of symbols (male: blue and light blue, female: pink and orange). Hormone treatments are indicated by shape of symbols (acetone treatment: circle, JHA treatment: square). RNAi treatments are distinguished by closed (*GFP* dsRNA injection) or open (*dsx* dsRNA injection). Scale bars indicate 10 mm (in males) or 5 mm (in females).

We predicted that sex-differences in tissue sensitivity to JH could be caused by action of the sex-determination cascade, specifically by expression of alternative splice variants of *Cmdsx*. To test for a functional role of *Cmdsx* in causing sex differences in the sensitivity of mandibular cells to JH, we applied a JH analog (JHA) to RNAi (*GFP* or *dsx*) treated males and females (Fig. 5). In control (*GFP* ^RNAi^) females, JHA application did not induce mandible growth (t = −0.611, *P* = 0.5549390199, Fig. 5), which corroborates our previously reported result that JHA application does not affect mandibular growth in wild-type females [5]. In contrast, JHA application to *dsx* ^RNAi^ females induced significant growth of mandibles compared with acetone application to *dsx* ^RNAi^ females (t = 2.254, *P* = 0.0429177662, Fig. 5). Thus, knockdown of *Cmdsx* caused mandibles of females to behave like those of males. Their growth became sensitive to JH, and therefore should also have become condition-dependent. In control (*GFP* ^RNAi^) males, JHA application induced significant mandible growth (t = 5.5500, *P* = 0.0004876470, Fig. 5), corroborating our earlier report that JHA application promotes mandibular growth in wild-type males [5]. For *dsx* ^RNAi^ males, JHA application rescued the defective mandibular phenotype by promoting mandibular growth (ANCOVA, t = 4.918, *P* = 0.0003321826, Fig. 5), however, the effect of JHA application tended to be decreased in *dsx* ^RNAi^ individuals. We suspect that here, too, the result was to make mandibles in males behave more like those of females. That is, growth of mandibles in knockdown males might be less sensitive to JH than it otherwise would have been.

## Discussion

### Doublesex and sex-specific trait expression

Spatiotemporal patterns of expression and functional analyses of *Cmdsx* support the hypothesis that sex-specific growth of exaggerated mandibles in stag beetles is controlled by *doublesex*. The differences in the 3′ end between male- and female-specific CmDsx are predicted to have important consequences for DNA binding. In *Drosophila*, it is known that this domain enhances DNA recognition by promoting dimerization of Dsx [35]. Thus, this difference of domain structure in CmDsx suggests differential DNA-binding ability of the predicted male and female proteins. In addition to the differences between sex-specific isoforms, all four isoforms differ from each other in their amino-acid sequence at the 3′ end, raising the possibility that each isoform is deployed differentially in space and time in a sex- and tissue-specific manner. Expression analyses in other body parts and isoform-specific knockdown experiments will be required to confirm this possibility.

Examination of *Cmdsx* expression in the sexually dimorphic mandibles compared to the sexually monomorphic maxillae revealed that there are differences in expression in a developmental and tissue specific pattern (Fig. 3C). Recent work in *Drosophila* showed that *dsx* expression was temporally and spatially restricted to body parts showing sexual dimorphism [36], [37]. In stag beetle mandibles, female-specific *Cmdsx* transcripts showed their highest levels of expression at the exact stage (Stage 3 of prepupal development) when mandibular cells proliferate maximally in males [5]. Thus, female specific *Cmdsx* expression coincides precisely with inhibition of mandibular cellular proliferation (Fig. 3C). Expression of male-specific *Cmdsx* transcripts in mandibles peaked just before this stage, during a period (prepupal Stage 2) when cells in male mandibles are especially sensitive to the growth-promoting effects of JH [5]. Both of these sex- and trait-specific patterns of expression are consistent with isoform-specific regulatory roles for *Cmdsx* during mandible growth. Considering the results of *Cmdsx* knockdown, we suggest that male-specific *Cmdsx* transcripts may promote mandible growth, and female-specific transcripts inhibit mandible growth, in part by enhancing or repressing the sensitivity of mandibular cells to JH.

### Doublesex and juvenile hormone signaling

Our results demonstrate, for the first time in any insect, a functional link between Dsx expression and JH signaling. Knockdown animals had significantly altered responses to topical JHA application, compared with control animals (Fig. 5). In females, knockdown of *Cmdsx* caused mandibles to be sensitive to JHA, where they otherwise would not have been, suggesting that normal expression of female-specific isoforms of CmDsx contributes to insensitivity of female mandibles to JH. Because JH acts to stimulate cell proliferation during this developmental stage, such a mechanism would repress excessive growth of this structure in females. In males, knockdown of *Cmdsx* combined with topical application of JHA stimulated some mandible growth, but not as much as in control animals with application of JHA. This indicates that male mandibles retained some sensitivity to JH even in their lowered expression of *Cmdsx*. We suggest that some sensitivity of mandibular cells to JH is the default developmental state for these animals. In normal males, male-specific CmDsx isoforms may increase the sensitivity of mandibular cells to JH, contributing to rapid and disproportionate growth of these exaggerated structures. In this case, knockdown of *Cmdsx* would remove this extra-sensitivity, restoring mandibular cells to their default state and producing males with large, but not extreme, mandible sizes. Another possibility is that *dsx* and JH act in parallel to regulate mandible growth in males. If this were the case, then *Cmdsx* and JH would act independently to stimulate exaggerated growth of male mandibles, and their effects would simply be additive. Thus, crosstalk between *dsx* and JH in males will need to be investigated in future studies.

Although there have been many previous reports of sex-specific JH actions on secondary-sexual characters in various insect lineages [38], [39], [40], [41], this study is the first to demonstrate a causal link between the JH signaling pathway and the sex determination pathway, which clearly interact to determine the developmental fates of secondary-sexual characters.

### The genetic and physiological mechanisms underlying sexually dimorphic traits

A recent study on *dsx* regulation of sexual dimorphism has also been reported for horned beetles (*Onthophagus taurus*) [20]. In this study, critical roles of *dsx* in sex differentiation, including development of sex-specific exaggerated traits, were shown. Horns in *O. taurus* are dimorphic in two ways: females do not produce horns (sexual dimorphism), and males smaller than a threshold body size produce only rudimentary horns (male dimorphism). Kijimoto et al showed that only large males expressed the male specific isoform of *Otdsx* (*OtdsxM*); small males did not [20]. Because body size and horn morphology depend critically on nutrition in this species, the findings of Kijimoto et al raise the possibility that levels of expression of *OtdsxM* may be sensitive to nutrition, as well as sex [20]. This contrasts with *C. metallifer*, where horns do not exhibit male dimorphism (all males produce enlarged mandibles) and where we find at best minimal evidence of nutrition-dependent expression of *dsx* (based on comparisons between large and small males). Expression of *Cmdsx* in mandibles of large males was at most 1.3 times that of mandibles in small males (during Stage 2; Fig. 3C), which is much smaller than the differences observed for *Onthophagus* (large males showed approximately 3 times higher expression of *OtdsxM* than small males). Based on these results we suggest that endocrine pathways sensitive to nutrition may interact with the sex determination pathway both upstream [20] and downstream (our study) of *dsx*.

### Additional developmental factors are likely involved in development of sexually dimorphic traits

**Figure 6:**
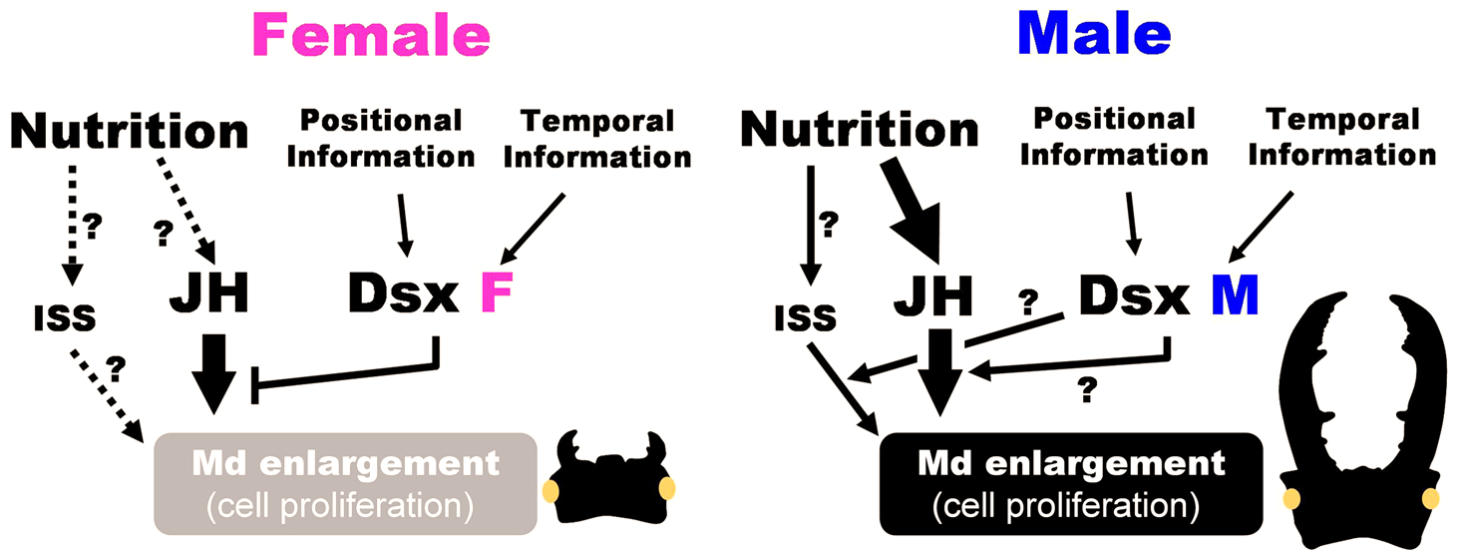
Schematic view of developmental link between nutrition and sex via JH signaling for sex-specific exaggerated trait development in the stag beetle. In addition to JH and Dsx, other possible factors were described which might be involved in integration of spatio-temporal information. Nutrition information is mediated by JH signaling and promote mandible enlargement in males. DsxM (CmDsxA and CmDsxB) might play a promoting role for JH-dependent mandible enlargement or recruiting other signaling pathway such as insulin signaling pathway (ISS). In females, DsxF (CmDsxC and CmDsxD) inhibit JH effect by reducing the JH sensitivity of mandible cell.

The inability of JHA treatment of *dsx* ^RNAi^ males to induce full growth of mandibles may indicate the action of other regulatory pathways for mandible growth. One likely candidate is the insulin-signaling pathway, because this pathway is known to regulate body and organ size in insects in accordance with nutritional conditions [42], [43], [44]. Growing horns in male rhinoceros beetles (*Trypoxylus dichotomus*) are known to be more sensitive to insulin signals than other metric traits (e.g., wings, genitalia) [3], and Emlen et al. reported sex- and morph- (major vs minor male) specific expression of the insulin receptor (InR) in growing horns of the dung beetle (*Onthophagus nigriventris*) [10]. It is likely that the enlarged mandibles of male stag beetles will also be sensitive to insulin signaling during their period of growth, and we suspect that *Cmdsx* may contribute to sex differences in sensitivity to these signals as well (Fig. 6). Future studies such as expression analyses of InR and insulin-like peptides will be needed to examine these additional mechanisms, but already it is clear that a rich interplay between endocrine and sex-determination pathways coordinates the growth of exaggerated sexually-selected and sexually-dimorphic characters.

## Materials and Methods

### Insect husbandry

Stag beetle adults (*Cyclommatus metallifer*) were purchased from Hercules-Hercules, Sapporo, Japan. Detail rearing and breeding methods are described in the Supporting Information (Text S1).

### Developmental staging of the prepupal period

We defined four developmental stages during the prepupal period based on specific developmental landmarks (Fig. 2). First, the stag beetle final instar larva constructs a pupal cell prior to pupation which marks the border between the end of the larval stage and the onset of the prepupal stage. After pupal cell construction (PCC), the larva undergoes a two-stage gut purge (GP) in which all gut contents are egested from the body. It takes two days from the onset of PCC to the start of the first GP, which is termed ‘Stage 1’ (Fig. 2). The period of time that the first GP continues lasts about 3–4 days and is ‘Stage 2’ (Fig. 2). Overall body weight gradually decreases during stage 2 (Fig. 2). After the first GP is over, the individual remains in a suspended state for 3–5 days during which time the adult structures are proliferating and growing (Stage 3). Stage 4 is a very brief period which corresponds just a few hours prior to pupation when the individual purges all of its remaining gut contents for the second GP and completes metamorphosis into the pupal stage.

### Cloning of *dsx* and reference genes from *C. metallifer*

Partial transcript sequences of the *C. metallifer* orthologs for *dsx* were cloned by degenerate PCR. Three additional transcripts for *C. metallifer* reference genes for real time qPCR were also cloned by degenerate PCR – *glyceraldehyde-3-phosphate dehydrogenase* (*GAPDH*), *elongation factor 1 alpha* (*EF-1a*) and *ribosomal protein L32* (*rpl32*). Primer sequences for degenerate PCR are listed in Table S1. Data base searches for homologies were performed using BlastX at the NCBI server (http://blast.ncbi.nlm.nih.gov/Blast.cgi). To further confirm the orthologues, we made multiple alignments of *dsx* genes including the orthologues from the other insect species, and constructed neighbor-joining trees of protein sequences (for *Cmdsx*) or mRNA sequences (for *GAPDH*, *EF-1a* and *rpl*32) using ClustalX program [45] (http://www.clustal.org/) (Fig. S3, S4, S5). Confidence was estimated with 1000bootstraps. Detailed cloning procedures are described in the Supporting Information (Text S1).

### Identify full-length of *dsx* gene by using RACE

Rapid amplification of cDNA ends (RACE) -PCR was performed to obtain the full length *C. metallifer dsx* transcript sequence using the following RACE primers (for 5′-RACE: 5′- CCT GAA CAC GTC GGG AAA AGA CGG CG-3′, for 3′-RACE: CTC GAA GAT TGC CAT AAG CTC CTG GAA AGG-3′) and the SMART RACE cDNA Amplification Kit (Clontech, Palp Alto, CA). The amplified cDNA fragments were subcloned and sequenced as described before. The protein domains were predicted by using CDD (conserved domain database) on NCBI (http://www.ncbi.nlm.nih.gov/Structure/cdd/wrpsb.cgi).

### RT-PCR and real-time quantitative PCR for *Cmdsx* expression analysis

The expression patterns of *Cmdsx* transcripts in small and large male and female prepupae were examined during the period of maximal mouthpart growth with RT-PCR and real-time qPCR. Briefly, individuals were reared under high versus low nutrition conditions that result in small versus large prepupae; details for this can be found in our previous study [5]. See Supporting Information (Text S1 and Table S2) for a detailed description of our methods for RT-PCR and real-time qPCR and primer sequences for real-time PCR.

### Knock-down of *dsx* by RNA interference

Functional analysis of the stag beetle *dsx* was accomplished by knockdown of the *dsx* transcript by RNA interference (RNAi) during prepupal development. To silence all *Cmdsx* transcripts, double-stranded RNA (dsRNA) against a 352 bp region of *Cmdsx* common to all four splice variants (Fig. 3A) was synthesized. Detailed procedures for dsRNA synthesis are described in the Supporting Information (Text S1). All dsRNA was diluted with 1×PBS. One µg of dsRNA in 5 µl of PBS was injected into the dorsal prothorax of late 3rd instar larvae using a microliter syringe (Hamilton, Reno, NV) under a stereomicroscope. This stage is just prior to the prepupal period and prior to adult mandibular cellular proliferation, so the effect of RNAi was targeted to pupal development (Fig. 2). Individuals that successfully eclosed into adults were used for analyses; these included 7 *GFP* ^RNAi^ females, 12 *dsx* ^RNAi^ females, 8 *GFP* ^RNAi^ males, and 16 *dsx* ^RNAi^ males.

For statistical test of *Cmdsx* RNAi effect, analysis of covariance (ANCOVA) was performed with body size as a covariate using R 3.0.1 software [46]. The equality of the slopes of regression lines was tested and no significant interaction was detected in female samples (F = 0.2779, P = 0.6058). In male samples, the slopes were significantly different between *GFP* ^RNAi^ and *dsx* ^RNAi^ samples (F = 19.072, P = 0.0002982).

RNAi efficiency was also examined by measurement of *Cmdsx* expression levels using real-time qPCR in the prepupal mandibles of males and females injected with dsRNA against *GFP* (control) or *Cmdsx*. Primers for real-time qPCR were designed to the common region shared by all isoforms (forward primer: 5′-TTC CGC TCT CAT TCA TAA ACGA-3′, reverse primer: 5′-TGC GGA AAA CGG CAA AGT-3′). To prevent overestimation of transcripts by detecting injected dsRNA, we designed the primers to amplify a region that had no overlap with the region used in dsRNA synthesis.

### JHA treatment on *GFP* and *dsx* RNAi individuals

To investigate the effects of *Cmdsx* on JH action, we combined a *Cmdsx* knockdown experiment with ectopic application of the JH analog (JHA). According to previous study [5], we used fenoxycarb for JHA application. First, we injected dsRNA against *GFP* or *Cmdsx* into the dorsal thorax of late 3rd instar *C. metallifer* larvae as described above. Then, when the knockdown experimental prepupae reached stage 2, five µg of the JH analog fenoxycarb diluted in 10 µl of acetone (Wako) was applied to the dorsal thorax according to previous study [5]. The control groups were dsRNA-injected pupae treated with acetone only. Pupal weight and pupal (in males) or adult (in females) mandible length were recorded. Sample sizes of surviving animals with normal, measurable traits are described in Table S3.

To estimate the effect of JHA application on relative mandible size for each of the four RNAi categories (*GFP* ^RNAi^ males, *dsx* ^RNAi^ males, *GFP* ^RNAi^ females, *dsx* ^RNAi^ females), analysis of covariance (ANCOVA) was performed with body size as a covariate using R 3.0.1 software [46]. The equality of the slopes of the regression lines was tested and no significant interaction was detected in all the four RNAi categories (F = 0.6789, P = 0.4313 in *GFP* ^RNAi^ males; F = 2.9354, P = 0.1029 in *dsx* ^RNAi^ males; F = 2.4669, P = 0.1507 in *GFP* ^RNAi^ females, F = 0.6751, P = 0.4185 in *dsx* ^RNAi^ females). Statistical significance of JHA application effects was adjusted for multiple comparisons by using Benjamini & Hochberg method [47].

## Supporting Information

Figure S1 Alignment of Dsx sequences. (A) Alignment of the conserved amino acid sequences of the predicted CmDsx protein and those of other insects. Only the two most conserved regions are presented and include the DNA binding (DM) domain and the dsx dimer domain (dashed black box). Identical amino acids are highlighted in black. Putative conserved residues that distinguish the Dsx DM domain from the DM domain of other proteins are shown by arrowheads. The dashed blue box indicates weak similarity of sequence among CmDsxA, CmDsxB and OtDsxM. The solid blue box indicates conserved 25 amino acid sequence in CmDsxB and OtDsxM. Cm: *Cyclommatus metallifer*, Tc: *Tribolium castaneum*, Bm: *Bombyx mori*, Aa: *Aedes aegypti*, Dm: *Drosophila melanogaster*, Ot: *Onthophagus taurus*, BmM: *Bombyx mori* male-type isoform, BmF: *Bombyx mori* female-type isoform. OtM: *Onthophagus taurus* male-type isoform, OtF1: *Onthophagus taurus* female-type isoform 1, OtF2: *Onthophagus taurus* female-type isoform 2 (B) A *dsx* gene tree based on the conserved DM domain amino acid sequences region using the neighbor-joining method with bootstrap support above the branches.
(TIF)

Figure S2 Effect of RNAi knockdown. Relative expression change of *Cmdsx* in prepupal *GFP* ^RNAi^ and *dsx* ^RNAi^ females (above) and males (below) in mandibles. The Y-axes show relative expression levels, which are specific to each panel. Averages and 95% confidence intervals of three technical replicates are indicated. Asterisks indicate significant differences between *dsx* ^RNAi^ and *GFP* ^RNAi^ samples (Student t-test with Bonferroni correction, P<0.0166).
(TIF)

Figure S3 Phylogenetic tree of GAPDH.
(TIF)

Figure S4 Phylogenetic tree of EF-1a.
(TIF)

Figure S5 Phylogenetic tree of RPL32.
(TIF)

Table S1 List of degenerate primer sequences for gene cloning.
(XLS)

Table S2 List of primer sequences for real-time qPCR.
(XLS)

Table S3 Sample sizes of surviving animals in combination experiment of dsx RNAi and JHA treatment.
(XLS)

Text S1 Supporting materials and methods.
(DOC)
