# Supplementary figures and images for: Developmental Link between Sex and Nutrition; doublesex Regulates Sex-Specific Mandible Growth via Juvenile Hormone Signaling in Stag Beetles
Source: PLoS Genet. 2014 Jan 16;10(1):e1004098. doi: 10.1371/journal.pgen.1004098 (PMC3894178; doi:10.1371/journal.pgen.1004098)

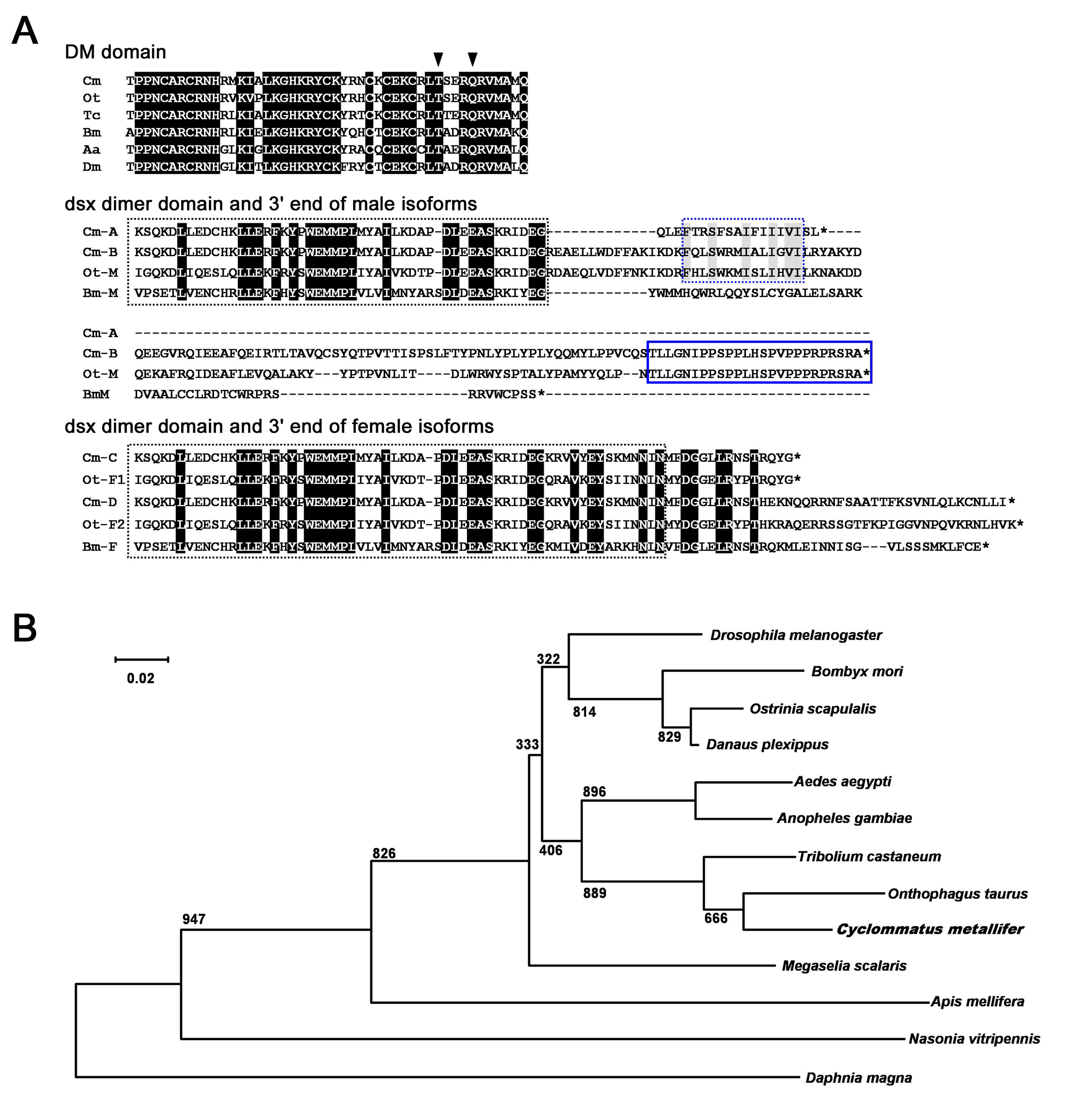

Supplement: Figure S1 — Alignment of Dsx sequences. (A) Alignment of the conserved amino acid sequences of the predicted CmDsx protein and those of other insects. Only the two most conserved regions are presented and include the DNA binding (DM) domain and the dsx dimer domain (dashed black box). Identical amino acids are highlighted in black. Putative conserved residues that distinguish the Dsx DM domain from the DM domain of other proteins are shown by arrowheads. The dashed blue box indicates weak similarity of sequence among CmDsxA, CmDsxB and OtDsxM. The solid blue box indicates conserved 25 amino acid sequence in CmDsxB and OtDsxM. Cm: Cyclommatus metallifer, Tc: Tribolium castaneum, Bm: Bombyx mori, Aa: Aedes aegypti, Dm: Drosophila melanogaster, Ot: Onthophagus taurus, BmM: Bombyx mori male-type isoform, BmF: Bombyx mori female-type isoform. OtM: Onthophagus taurus male-type isoform, OtF1: Onthophagus taurus female-type isoform 1, OtF2: Onthophagus taurus female-type isoform 2 (B) A dsx gene tree based on the conserved DM domain amino acid sequences region using the neighbor-joining method with bootstrap support above the branches. (TIF) [file pgen.1004098.s001.tif]

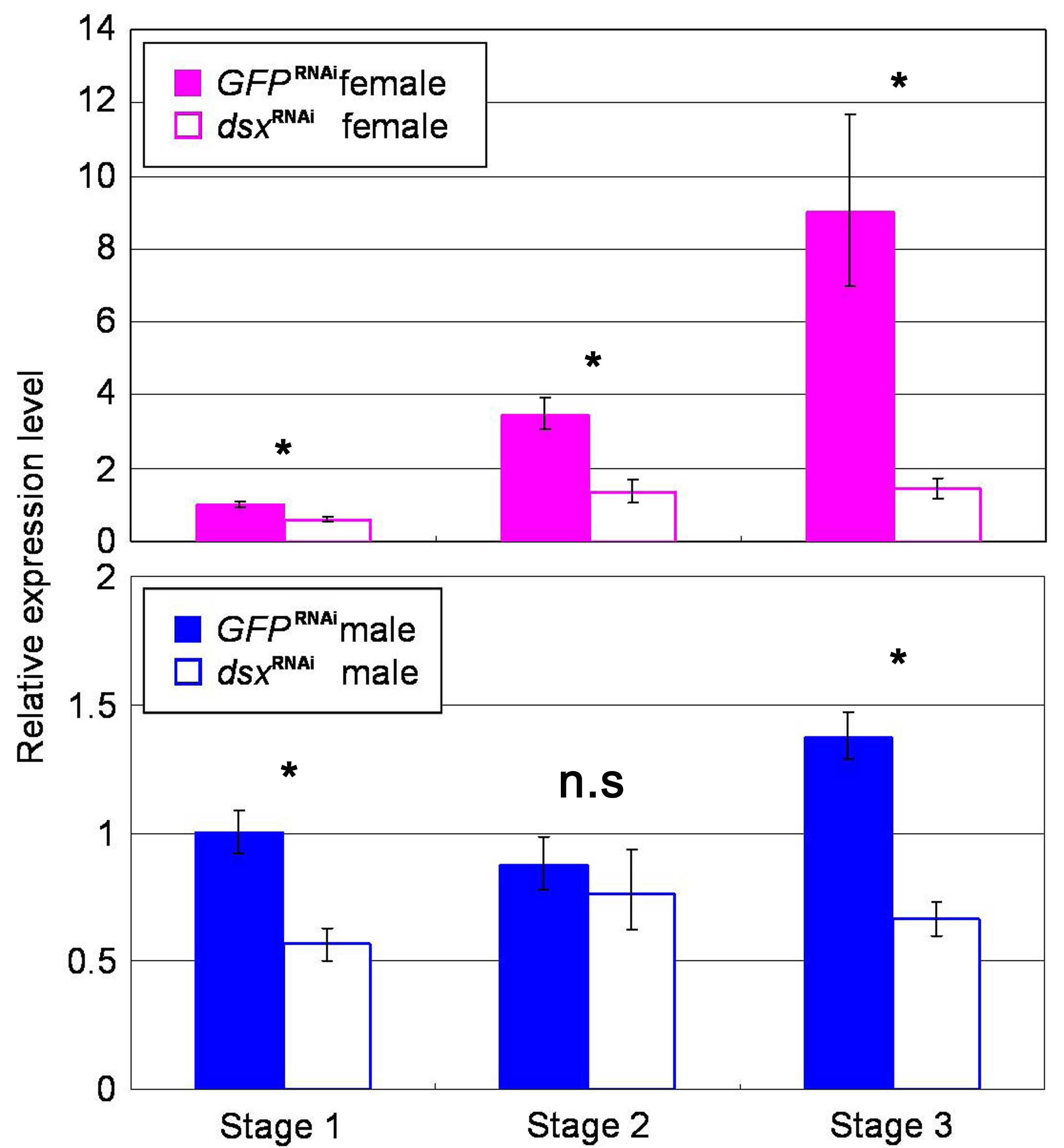

Supplement: Figure S2 — Effect of RNAi knockdown. Relative expression change of Cmdsx in prepupal GFP RNAi and dsx RNAi females (above) and males (below) in mandibles. The Y-axes show relative expression levels, which are specific to each panel. Averages and 95% confidence intervals of three technical replicates are indicated. Asterisks indicate significant differences between dsx RNAi and GFP RNAi samples (Student t-test with Bonferroni correction, P<0.0166). (TIF) [file pgen.1004098.s002.tif]

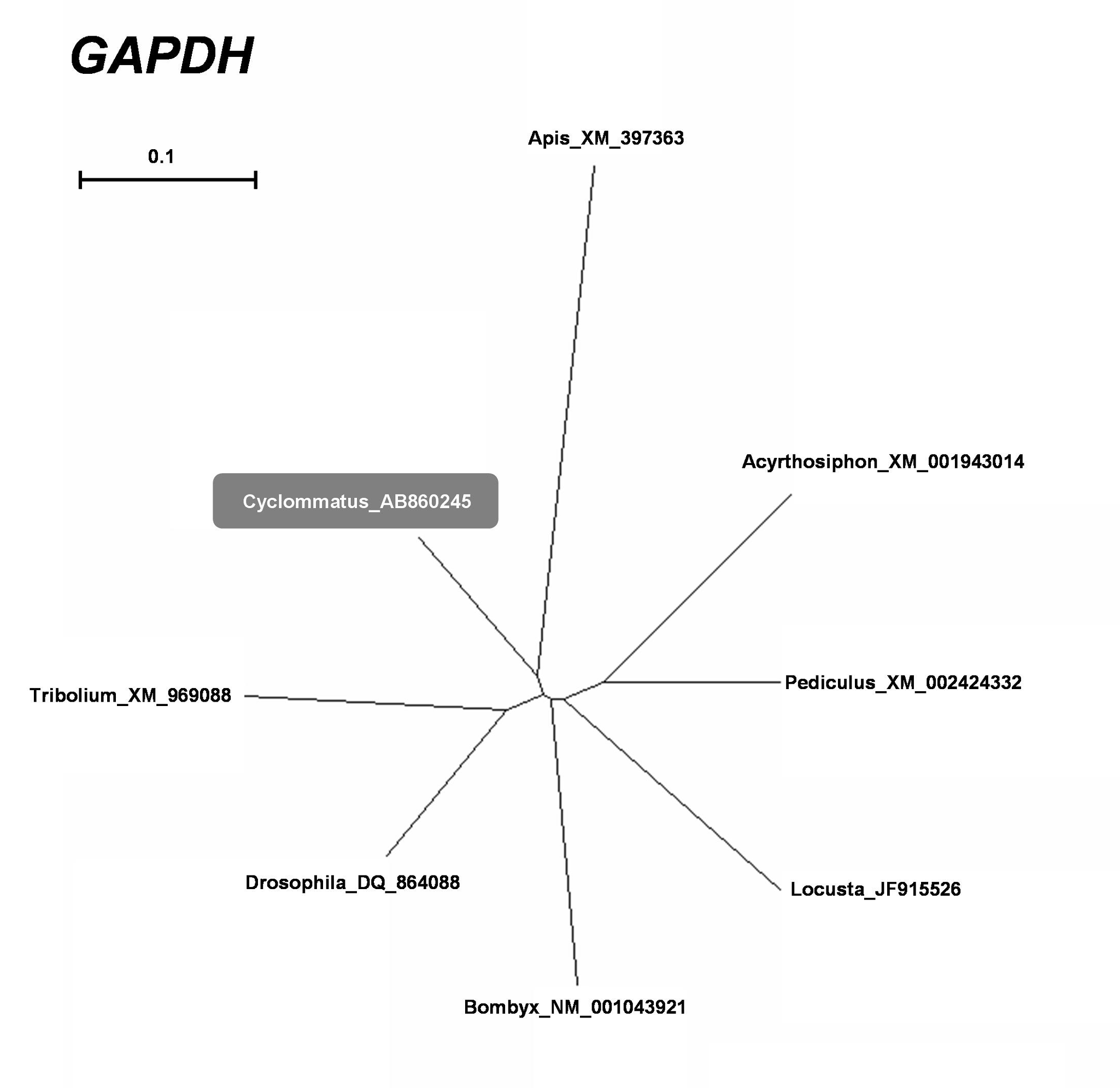

Supplement: Figure S3 — Phylogenetic tree of GAPDH. (TIF) [file pgen.1004098.s003.tif]

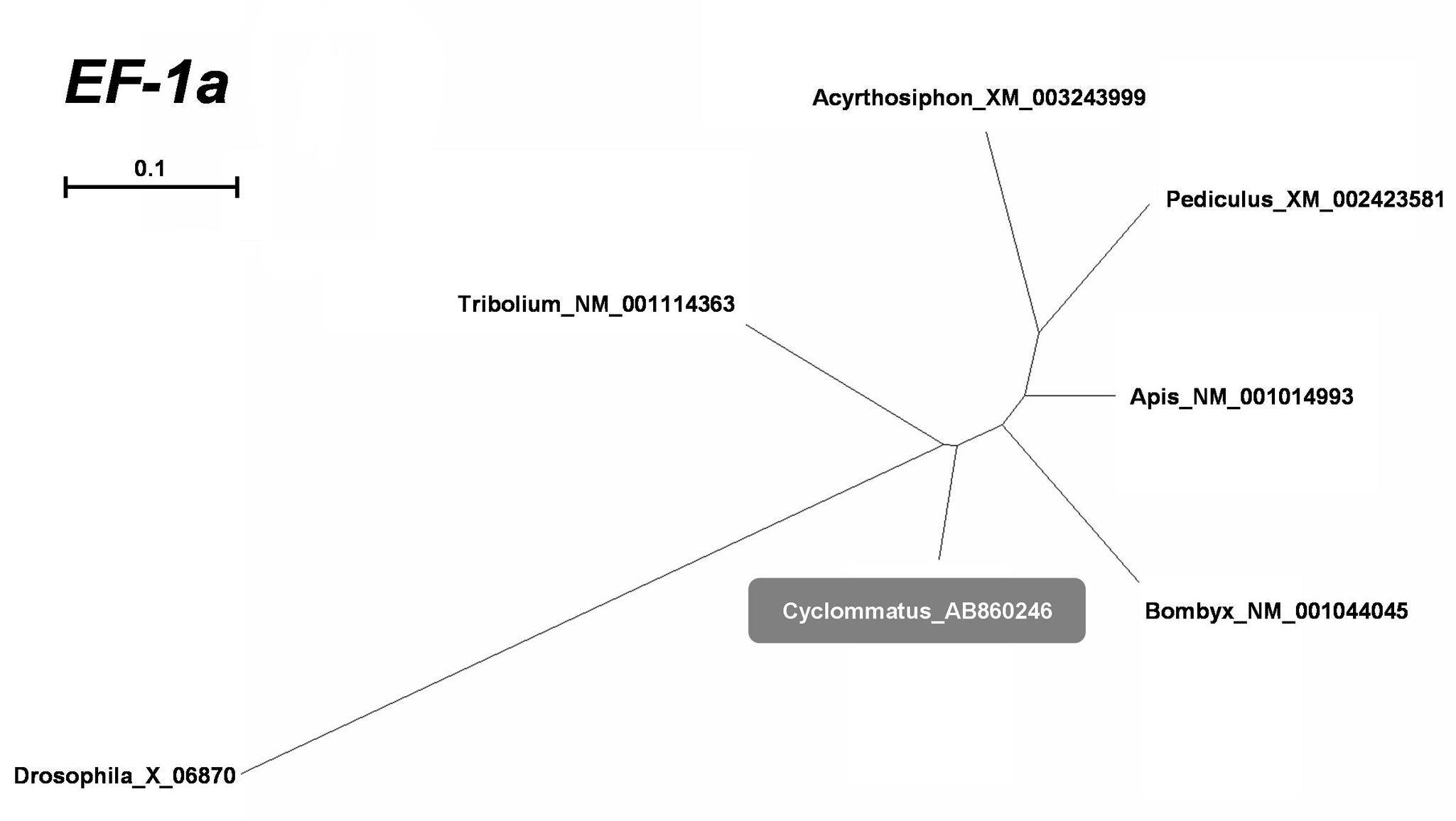

Supplement: Figure S4 — Phylogenetic tree of EF-1a. (TIF) [file pgen.1004098.s004.tif]

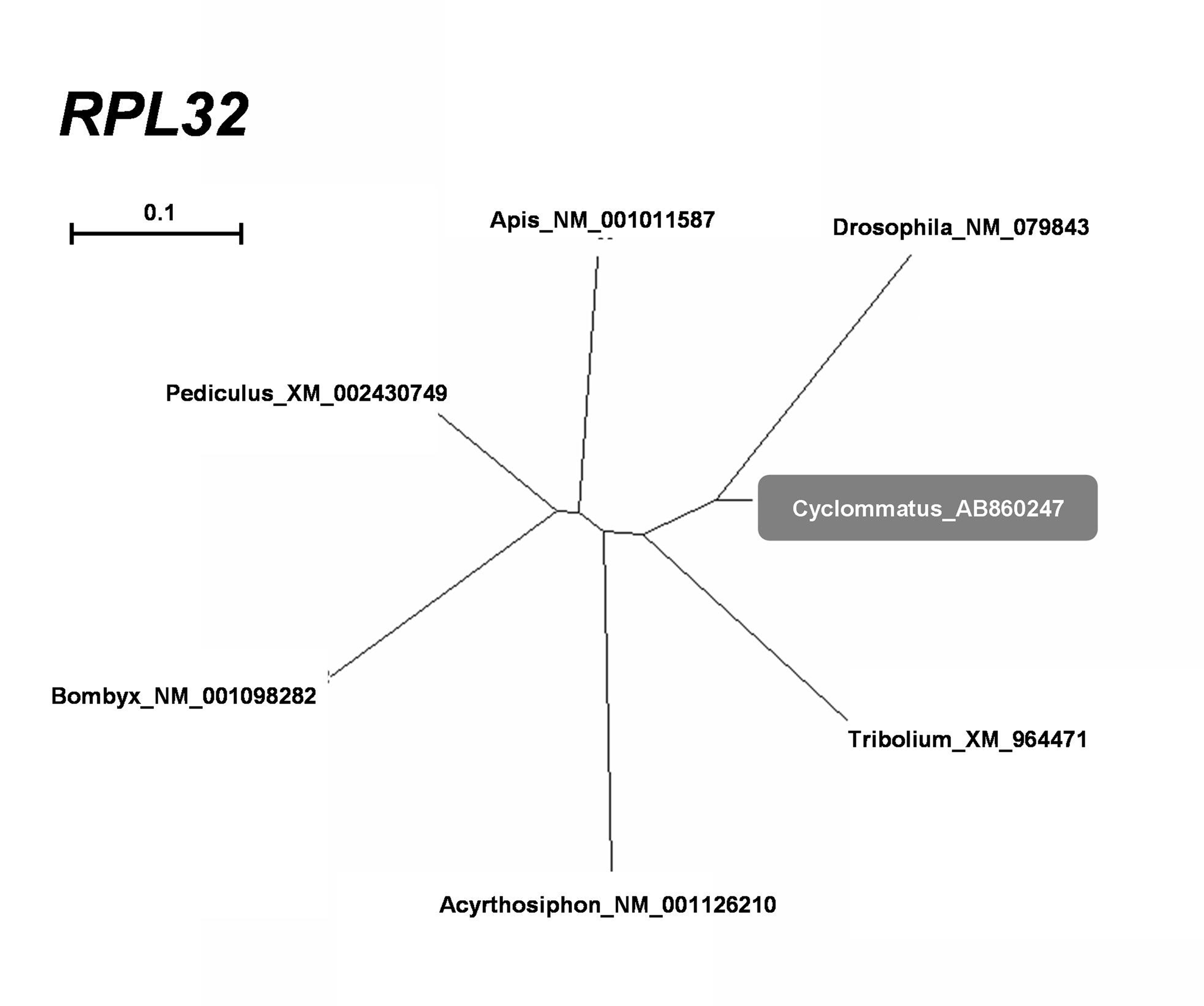

Supplement: Figure S5 — Phylogenetic tree of RPL32. (TIF) [file pgen.1004098.s005.tif]
