# Supplementary material for: Developmental Link between Sex and Nutrition; doublesex Regulates Sex-Specific Mandible Growth via Juvenile Hormone Signaling in Stag Beetles
Source: PLoS Genet. 2014 Jan 16;10(1):e1004098. doi: 10.1371/journal.pgen.1004098 (PMC3894178; doi:10.1371/journal.pgen.1004098)
Supplement: Text S1 — Supporting materials and methods. (DOC) [file pgen.1004098.s009.doc]

**Text S1, Supporting Materials and Methods**

*Insect Husbandry*

All adults were reared and bred in the laboratory under 24 hour darkness at 24 ± 2ºC. To breed these beetles, one male and one to two females were placed together in an 850 ml plastic container containing approximately 600 ml of decaying wood flakes (genus *Quercus;* Kuwagata-mat, Hercules-Hercules). Adults were fed with a commercial beetle jelly (Za-Okuwa Co., Kawanishi, Japan) supplied once a week. After two months, adults were removed and eggs and larvae were placed into a 27 cm × 35 cm × 21.5 cm plastic container filled with decaying wood flakes. The eggs and larvae were reared in mass culture under 24 hour darkness at 24 ± 2 °C until the second instar when they were removed, individually maintained, and observed for exact developmental staging of the final instar.

*Cloning of dsx and reference genes from C. metallifer*

Partial transcript sequences of the *C. metallifer* orthologs for *dsx* was cloned by degenerate PCR. Three additional transcripts for *C. metallifer* reference genes for quantitative real time PCR were also cloned by degenerate PCR -- *glyceraldehyde-3-phosphate dehydrogenase* (GAPDH), *elongation factor 1 alpha* (EF-1a) and *ribosomal protein L32* (RPL32). The heads were removed from two male and two female larvae, frozen in liquid nitrogen, and preserved at –80°C until RNA extraction. Total RNA was extracted using the SV total RNA Isolation System (Promega, Madison, WI) according to the manufacturer’s protocol. For each sample, 2 μg of total RNA was reverse transcribed with the High Capacity cDNA Reverse Transcription Kit according to the manufacturer’s instructions (Applied Biosystems, Foster City, CA). Using the reverse-transcribed cDNA as template, gene fragments were obtained by PCR amplification with specific degenerate primers (Supplementary Table 1) and with Ex-Taq polymerase mix (Takara Bio., Ohtsu, Japan). The amplified fragments were electrophoresed and the candidate bands were purified with the Wizard SV Gel and PCR Clean-up System (Promega) and ligated into the pGEM-T vector System (Promega). Ligated plasmids were transfected in E. coli JM 109 (Takara Bio), and were cultured at 37°C for 16 hours, then purified with Sigma Gene Elute Plasmid Miniprep Kit (Sigma-Aldrich, St. Louis, MO). The nucleotide sequence of each insert cDNA fragment was determined with a Dye Terminator cycle sequencing kit and automatic sequencer model 3100 (Applied Biosystems). Data base searches for homologies were performed using BlastX at the NCBI server (http://blast.ncbi.nlm.nih.gov/Blast.cgi). To further confirm the orthologues, we aligned our putative *dsx* genes with other identified genes from other animals, including insects, and constructed neighbor-joining trees of protein sequences (for *dsx*) or mRNA sequences (for *GAPDH, EF-1a, RPL32*) using ClustalX ([45]; http://www.clustal.org/). The resulting topologies were bootstrapped 1000 times to estimate confidence in the nodes.

.

*RT-PCR and Real-time quantitative PCR for Cmdsx expression analysis*

For each of the four developmental stages, five prepupae of each sex were dissected. Mandibles and maxillae were isolated from all 20 individuals, frozen in liquid nitrogen, and preserved at -80°C. Total RNA was extracted with SV total RNA Isolation System (Promega) according to the manufacturer’s protocol. For each sample, 800 ng of total RNA was reverse transcribed with the High Capacity cDNA Reverse Transcription Kit according to the manufacturer instructions (Applied Biosystems). These synthesized cDNAs were used as templates for both RT-PCR and real-time qPCR described as follows. RT-PCR was performed with a forward primer (F1: 5’-CTC GAA GAT TGC CAT AAG CTC CTG GAA AGG–3’) and the exon-specific reverse primers (R1: 5’-GGC TAG AAC GCA AAG AAA CTA AAA TTA–3’, R2: 5’-GCG GGC TTG GTG GTA TGT T–3’, see Fig. 3A for the primer positions) under the following conditions: 94°C for 2 min; 35 cycles of 94°C for 30s, 55°C for 1min and 72°C for 3 min; and 72°C for 10 min, by using LA taq (Takara Bio). GAPDH was used as positive control (forward primer: 5’-GGG CGC CAA AAG GGT TAT T–3’, reverse primer: 5’-AAC AAA CAT TGG CGC ATC AG–3’, amplified product was 55 bp). Relative quantification of cDNAs was performed by real-time qPCR using Fast SYBR Green Master Mix (Applied Biosystems) and sequence detection system ABI PRISM 7500 (Applied Biosystems). For determining endogenous control of constitutive expression, the suitability of different putative reference genes was first evaluated. Using the softwere geNorm [48], *GAPDH* was found to be the most appropriate reference gene for comparison among categories. By using Primer Express software (Applied Biosystems), primers for both target and reference genes were designed (Supplementary Table 2). Data acquisition and analysis were handled by ABI Prism 7500 software v2.0.1 (Applied Biosystems). Baselines and thresholds for Ct were set automatically and *dsx* expression levels were normalized to *GAPDH* expression. We set normalized *dsx* expression level of Stage 1 female mandible (Exon 1) or Stage 2 female mandible (Exon 4, 6, 8, 9) as 1.0 for comparing to other columns. The significant expression differences among the categories were evaluated by the Tukey–Kramer multiple comparison test (*P* < 0.05) using Excel statistics software (Social Survey Research Information, Tokyo, Japan).

*dsRNA synthesis for RNAi*

Double-stranded RNA (dsRNA) against a 352 bp region of *Cmdsx* common to all four splice variants (Fig. 3A) was used for synthesizing dsRNA in order to silence all *Cmdsx* transcripts. A plasmid containing this region was cut by restriction enzyme Not I (Toyobo, Osaka, Japan) to obtain single-strand plasmids. With these purified plasmids and the MEGAscript RNAi Kit (Ambion, Austin, TX), synthesis of single-stranded RNA were performed, followed by annealing and purification of dsRNA. Synthesis of dsRNA against green fluorescent protein (GFP) was conducted using the method described for *Cmdsx* using GFP pQBI-polII (Wako, Osaka, Japan) as the template.

References

48. Vandesompele J, De Preter K, Pattyn F, Poppe B, Van Roy N, et al. (2002) Accurate normalization of real-time quantitative RT-PCR data by geometric averaging of multiple internal control genes. Genome Biol 3: research0034.1-0034.11.
